# Complete Genome Sequencing of a Classical Swine Fever Virus Strain Endemic in Vietnam

**DOI:** 10.1128/genomeA.00307-18

**Published:** 2018-05-03

**Authors:** Ha Thanh Tran, Hoang Vu Dang, Dung Tien Nguyen, Kohtaro Miyazawa, Takehiro Kokuho

**Affiliations:** a Department of Biochemistry and Immunology, National Institute of Veterinary Research, Hanoi, Vietnam; b Vietnam Veterinary Association, Hanoi, Vietnam; c Department of Virus Research, National Institute of Animal Health, National Agriculture and Food Research Organization, Tsukuba, Japan; d Department of Animal Disease Control and Prevention, National Institute of Animal Health, National Agriculture and Food Research Organization, Tsukuba, Japan

## Abstract

A Vietnamese strain of classical swine fever virus, VN91, was isolated in Hung Yen in 1991. While VN91 has been used as a challenge strain in efficacy tests of vaccines, its genetic background has never been described. Here, we report the genome sequence of the strain circulating in Vietnam.

## GENOME ANNOUNCEMENT

Classical swine fever virus (CSFV) is an enveloped single-stranded positive-sense RNA virus belonging to the genus Pestivirus. CSFV is one of the most important transboundary disease viruses in both domestic and international animal hygiene due to its virulence and contagiousness, which may cause serious problems in pig production. In Vietnam, CSF has been reported nationwide, and in some cases, the virus was isolated from infected pigs. However, insufficient vaccination against and a lack of genetic information about viruses endemic in Vietnam impede an understanding of the status of virus circulation in the country. For better understanding of the status, in this study, we attempted to determine the whole-genome sequence of a strain endemic in Vietnam, VN91, isolated from a pig that died from CSF during the outbreak in Hung Yen province in 1991. This highly pathogenic virus endemic in Vietnam has been used as a challenge strain for efficacy tests of commercial vaccines sold in Vietnam (1); however, its genetic information has not yet been analyzed. The VN91 strain was propagated in the culture of PK15a cells for 4 to 5 days at 37°C, and total RNAs were extracted from the culture supernatant. The RNAs were subjected to a reverse transcription reaction with the use of random oligonucleotide primers and were amplified by PCR with various sets of primers designed based on the genome sequences of previously known CSFV strains to prepare 25 overlapping cDNA fragments spanning the entire lengths of the genome for sequencing analysis. Determination of the 5′- and 3′-termini of the genome were performed by PCR in combination with the ligation of a 5′-phosphorylated oligonucleotide tag, as reported by Troutt et al. (2) and Becher et al. (3), with slight modification in the tag sequence. The VN91 strain had a genome of 12,298 bp in size and was classified into genotype 1.1 in terms of the nucleotide sequence similarities in the 5′-end untranslated region (UTR) and E2 region with those of other strains in this lineage by phylogenic tree analysis (4, 5). The VN91 strain indicated 72.0 to 99.0% genetic similarities with other viruses belonging to genotype 1.1, showing a strikingly high similarity (99.0%) with an attenuated vaccine strain, HCLV (GenBank accession no. AF091507).

As far as we know, this is the first report on the genome information of a CSFV of Vietnamese origin. In Vietnam, there are currently four different CSFV vaccines, a homemade one using the lapinized attenuated Chinese strain named Dong Kho dich ta heo (Navetco, Vietnam), Coglapest (Ceva, Hungary), Pest-Vac (Fort Dodge, Brazil), and Pestiffa (Merial, France), all of which belong to genotype 1.1 (6), rendering the discrimination of these vaccine strains from pathogenic field isolates difficult. The genomic sequence data of endemic strains of CSFV like the one we present here would be valuable for clarifying the situation on CSFV circulation, as well as for effective implementation of preventive measures against the disease in Vietnam.

### Accession number(s).

The genome sequence of the VN91 strain has been submitted to DDBJ/EMBL/GenBank under the accession no. LC374604.
